# Multi-omic analysis identifies hypoalbuminemia as independent biomarker of poor outcome upon PD-1 blockade in metastatic melanoma

**DOI:** 10.1038/s41598-024-61150-y

**Published:** 2024-05-16

**Authors:** Lindsay V. M. Leek, Jessica C. L. Notohardjo, Karlijn de Joode, Eline L. Velker, John B. A. G. Haanen, Karijn P. M. Suijkerbuijk, Maureen J. B. Aarts, Jan Willem B. de Groot, Ellen Kapiteijn, Franchette W. P. J. van den Berkmortel, Hans M. Westgeest, Tanja D. de Gruijl, Valesca P. Retel, Edwin Cuppen, Astrid A. M. van der Veldt, Mariette Labots, Emile E. Voest, Joris van de Haar, Alfons J. M. van den Eertwegh

**Affiliations:** 1 Department of Medical Oncology, Netherlands Cancer Institute, Antoni Van Leeuwenhoek, Amsterdam, The Netherlands; 2 Department of Medical Oncology, Amsterdam UMC Location Vrije Universiteit, Amsterdam, The Netherlands; 3 Department of Medical Oncology and Radiology & Nuclear Medicine, Erasmus MC Cancer Institute, University Medical Center, Rotterdam, The Netherlands; 4 Department of Medical Oncology, UMC Utrecht Cancer Center, Utrecht University, Utrecht, The Netherlands; 5 Department of Medical Oncology, GROW School for Oncology and Developmental Biology, Maastricht University Medical Centre, Maastricht, The Netherlands; 6 Department of Medical Oncology, Oncology Center Isala, Isala, Zwolle, The Netherlands; 7 Department of Medical Oncology, Leiden University Medical Centre, Leiden, The Netherlands; 8 Department of Medical Oncology, Zuyderland Medical Centre, Sittard-Geleen, The Netherlands; 9 Department of Medical Oncology, Amphia Hospital, Breda, The Netherlands; 10 Division of Psychosocial Research and Epidemiology, Netherlands Cancer Institute-Antoni Van Leeuwenhoek, Amsterdam, The Netherlands; 11 Health Technology and Services Research Department, Technical Medical Centre, University of Twente, Enschede, The Netherlands; 12 Hartwig Medical Foundation, Amsterdam, The Netherlands; 13 Center for Molecular Medicine and Oncode Institute, University Medical Center Utrecht, Utrecht, The Netherlands

**Keywords:** Prognostic markers, Melanoma

## Abstract

We evaluated the prognostic value of hypoalbuminemia in context of various biomarkers at baseline, including clinical, genomic, transcriptomic, and blood-based markers, in patients with metastatic melanoma treated with anti-PD-1 monotherapy or anti-PD-1/anti-CTLA-4 combination therapy (n = 178). An independent validation cohort (n = 79) was used to validate the performance of hypoalbuminemia compared to serum LDH (lactate dehydrogenase) levels. Pre-treatment hypoalbuminemia emerged as the strongest predictor of poor outcome for both OS (HR = 4.01, 95% CI 2.10–7.67, Cox *P* = 2.63e−05) and PFS (HR = 3.72, 95% CI 2.06–6.73, Cox *P* = 1.38e−05) in univariate analysis. In multivariate analysis, the association of hypoalbuminemia with PFS was independent of serum LDH, IFN-γ signature expression, TMB, age, ECOG PS, treatment line, treatment type (combination or monotherapy), brain and liver metastasis (HR = 2.76, 95% CI 1.24–6.13, Cox *P* = 0.0131). Our validation cohort confirmed the prognostic power of hypoalbuminemia for OS (HR = 1.98, 95% CI 1.16–3.38; Cox *P* = 0.0127) and was complementary to serum LDH in analyses for both OS (LDH-adjusted HR = 2.12, 95% CI 1.2–3.72, Cox *P* = 0.00925) and PFS (LDH-adjusted HR = 1.91, 95% CI 1.08–3.38, Cox *P* = 0.0261). In conclusion, pretreatment hypoalbuminemia was a powerful predictor of outcome in ICI in melanoma and showed remarkable complementarity to previously established biomarkers, including high LDH.

## Introduction

Immune checkpoint inhibitors (ICI) have induced clinical responses of unprecedented durability, transforming the standard of care for patients with metastatic melanoma^1–5^. However, durable responses are only observed in 40–60% of patients with metastatic melanoma who receive anti-programmed cell death protein 1 (anti-PD-1) monotherapy or anti-PD-1/anti-cytotoxic T-lymphocyte-associated protein 4 (anti-CTLA-4) combination therapy, highlighting the need for highly specific biomarkers to identify ICI-resistant patients and prevent overtreatment^6^*.* Over the past few years, a set of standalone biomarkers has been described to determine the response and resistance to ICI across cancer types^7^. Several studies identified high tumor mutational burden (TMB)^8^, T-cell infiltration^9^, PD-1 expression, programmed death-ligand 1 (PD-L1) expression^10^ and interferon-gamma (IFN-γ) signature expression^11^ at baseline (pre-immunotherapy) as potential predictors of response to immunotherapy. In contrast, poor outcome was associated with high serum LDH levels^12^, high serum neutrophil-lymphocyte-ratio (NLR)^13^, and, more recently, hypoalbuminemia^14–16^. These biomarkers offer valuable information that may assist clinicians in tailoring personalized treatment strategies and ultimately improve clinical outcomes for patients.

Traditionally, serum albumin has been viewed as a general marker for patients’ performance status and disease burden, providing insight into the close relationship between serum albumin levels and metabolic activity, nutritional status, and (systemic) inflammation^17^. In the context of cancer, hypoalbuminemia has been linked to unfavorable prognosis in numerous malignancies, either as a standalone marker or as part of a broader set of blood measurements (e.g., C-reactive protein, NLR, and LDH)^18–21^. Recently, evidence has been accumulating that hypoalbuminemia might serve as a potent, pan-cancer biomarker for poor response to ICI therapy with high complementarity to TMB^14,15^ and features derived from medical images and digital pathology in a multimodal biomarker study in NSCLC^16^. In melanoma, however, it remains unclear how serum albumin levels relate to other published biomarkers, including LDH, TMB, IFN-γ signature expression, and what added benefit this readily available and cost-effective biomarker may offer when incorporated into multimodal biomarker approaches.

Building on this literature, we hypothesized that hypoalbuminemia would also be of value in multimodal biomarker models to guide ICI treatment in melanoma. To investigate this concept, we collected a comprehensive set of whole-genome sequencing (WGS), RNA sequencing (RNA-seq), serum albumin and other blood-based laboratory measurements, and clinical data. A multimodal set of biomarkers was then tested for their complementarity and redundancy with hypoalbuminemia for the prediction of ICI treatment outcomes in metastatic melanoma.

## Materials and methods

### Population and study procedures

We collected data from 178 patients with metastatic melanoma who received systemic anti-PD-1 monotherapy (nivolumab, *n* = 54; pembrolizumab, *n* = 70) or anti-PD-1/anti-CTLA-4 combination therapy (nivolumab plus ipilimumab, *n* = 54) and had undergone a biopsy as part of the Center for Personalized Cancer Treatment (CPCT-02) study (NCT01855477) (Supplementary Table S1)^22^. Given that patients received either mono- or combination therapy, all multivariate analyses were corrected for treatment type, or alternatively, performed exclusively on those patients who received monotherapy. Patients were included from nine Dutch medical centers from April 2016 to December 2019 and were followed until 26 February 2021 (median: 36.8 months, range: 8.8–57.1 months). Only patients with evaluable treatment responses were included in the analyses. For validation, we analyzed an additional cohort, consisting of 79 patients with metastatic melanoma treated with monotherapy or combination therapy at Amsterdam UMC.

### Ethics declarations

The CPCT-02 study was conducted in accordance with the Declaration of Helsinki and Good Clinical Practice guidelines and was approved by the institutional review board of University Medical Center Utrecht. All patients provided written informed consent for WGS and data sharing for cancer research purposes. The validation cohort was approved by the medical ethical committee and was not deemed subject to the Medical Research Involving Human Subjects Act in compliance with Dutch regulations (2019.682).

### Study outcomes and biomarkers

Overall survival (OS) was defined as the time from the start of ICI treatment to death (event) or last follow-up (censored). Progression-free survival (PFS) was defined as the time from initiation of ICI treatment to the date of evaluated disease progression or death (event) or last follow-up (censored). Durable clinical benefit (DCB) was defined as either complete response (CR)/partial response (PR) or stable disease (SD) for at least 6 months, whereas no durable clinical benefit (NCB) was defined as progressive disease (PD) within 6 months from the start of ICI treatment, according to Response Evaluation Criteria in Solid Tumor (RECIST version 1.1)^23^.

Pretreatment genomics data were prospectively collected, and mutation and copy number calling were performed as previously described^22^. Genomic variables considered as (potential) biomarkers included the TML, structural variant load, whole-genome duplication, ploidy status, polyclonal proportion, and sequencing-based tumor purity^22^. Complementary clinicopathologic data were collected and, depending on availability, included date of birth, sex, age, Easter Cooperative Oncology Group performance status (ECOG PS), anatomical biopsy location, (number of) prior treatments, anatomical metastatic site, date of progression/death/last follow-up, and blood-based laboratory measurements including hemoglobin, white blood cells, platelets, NLR, monocytes, eosinophils, basophils, LDH, albumin, and tumor marker S100. The upper and lower limits of normal were defined based on the clinical standards of the coordinating institute and are listed in Supplementary Table S2.

TMB was defined as the total number of mutations and small indels per mega base genome-wide, with > 10 mutations per mega base (Mb) representing a high TMB; this cutoff was FDA-approved for anti-PD-1 therapy^24^. The probability of active ultraviolet (UV) mutational signature indicates UV-based mutagenesis with the probability threshold set at > 0.5^25^. All mutations were annotated with SNPeff and SNPsift v5.0e and were either oncogenes or biallelic tumor suppressor genes, classified as moderate to high impact^26^. A selection of mutations, present in at least 5 patients, were tested for association with treatment outcomes (Supplementary Table S3). This selection included driver mutations, and mutations were included when found in key genes that are involved in major histocompatibility complex (MHC) folding and presentation, antigen processing, and insensitivity to IFN-γ signaling. Driver mutations were called using PURPLE v3.7.1 as previously described (driver likelihood > 0.5)^22^.

For RNA-seq-based analyses, several expression signatures were derived, including the IFN-γ signature gene set^11^, tumor-infiltrating lymphocytes (TILs) signature^27^, and a collection of immune checkpoints^28^ (Supplementary Table S4) and hallmark gene sets (MSigDB)^28–30^. The cutoff of expression-based features was determined by identifying the intersection of each variable between DCB and NCB, and for instances where multiple intersections were present, the one closest to the mean was selected.

### Identification of biomarkers

First, previously published biomarkers were tested for their association with outcome in our cohort using univariate analysis, to select candidates for inclusion in multivariate modeling. Univariate Cox proportional hazard (Cox PH) regression was deployed to detect associations with OS and PFS, and Fisher’s exact test was used for links with DCB. Next, correlations among validated biomarkers were assessed to mitigate redundancy and collinearity. A single biomarker was selected from each cluster of biomarkers with Pearson correlations ≥ 0.5, based on the following rationales. TMB was selected as the representative for the correlated DNA features as this mirrors the neoantigen load and is known as an FDA-approved biomarker for several cancers^24^. As IFN-γ signature is the most well-established RNA-based biomarker for ICI in melanoma and has been widely described and validated in literature, this biomarker was therefore prioritized over the other, highly correlated RNA-based features^11,31,32^. Biomarkers exhibiting a proportion of missing data exceeding 30% were omitted from multivariate modeling, thereby excluding S100 and NLR markers with a percentage of missing data of 56% and 49%, respectively. Only patients with all data points present for the relevant biomarkers were considered for multivariate analyses (*n* = 85). Multivariate Cox PH regression was performed on OS and PFS to evaluate whether the noncollinear biomarkers held independent prognostic capacity for treatment outcomes. Proportion of variance explained was calculated as Pearson correlation coefficient^2^ × 100 between pairs.

### Statistical procedures

Statistical analyses were performed using R software, version 4.0.3 (https://www.r-project.org). Univariate and multivariate associations of each biomarker with OS and PFS were tested by the Cox PH regression model with a reported hazard ratio (HR), a corresponding 95% confidence interval (CI). All tests were two-sided, and a *P* smaller than or equal to 0.05 were considered statistically significant. Pearson’s chi-squared test and Fisher’s exact test assessed the significance of a difference between the proportions of DCB and NCB in the high versus low groups per biomarker. In the explorative analyses on mutations and RNA signatures, *P* was False Discovery Rate (FDR)-corrected using the Benjamini–Hochberg procedure.

## Results

### Clinico-pathological characteristics

**Figure 1:**
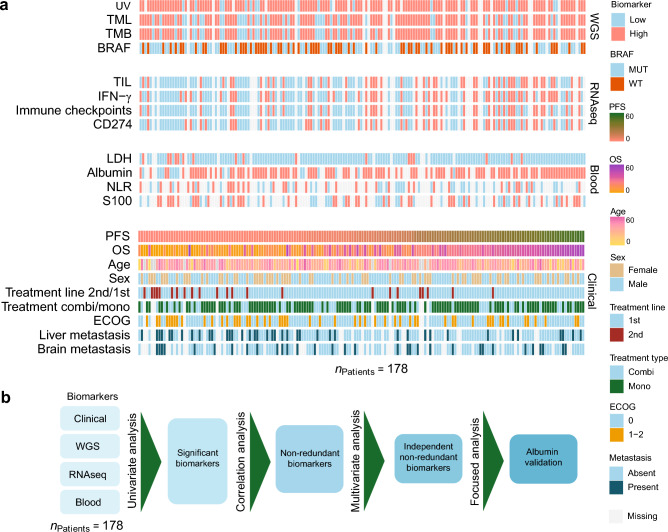
Overview of multimodal biomarkers and study design. (**a**) Heatmap showing patient characteristics and pretreatment biomarkers for ICI–treated metastatic melanoma patients, grouped from top to bottom in clinical, WGS, RNA-seq, and blood-derived measures. Each column represents pretreatment information for a single patient. Missing values are shown in gray. (**b**) Flowchart outlining the steps of methods. Abbreviations: *BRAF*, v-Raf murine sarcoma viral oncogene homolog B1; combi, combination therapy; DCB, durable clinical benefit; ECOG PS, Eastern Cooperative Oncology Group performance status; HR, hazard ratio; ICI, immune checkpoint inhibitor; IFN-γ, interferon-gamma; LDH, lactate dehydrogenase; mono, monotherapy; MUT, mutation; mts, metastasis; NA, not available; NLR, neutrophil-lymphocyte-ratio; RNA-seq, RNA sequencing; S100, serum S100 melanoma marker; TIL, tumor-infiltrating lymphocytes; TMB, tumor mutation burden; TMB, tumor mutational burden; UV, ultraviolet mutational signature; WGS, whole-genome sequencing; WT, wild-type.

We collected WGS, RNA-seq, blood-based laboratory measurements, and real-world clinical data of 178 patients with metastatic cutaneous melanoma treated with anti-PD-1 monotherapy (nivolumab, *n* = 54; pembrolizumab, *n* = 70) or combined anti-PD-1/anti-CTLA-4 (nivolumab plus ipilimumab, *n* = 54) (Fig. 1a; Table 1). The workflow is depicted in Fig. 1b. Across the total study population, the median PFS and OS were 8 (range: 0–51 months) and 21 months (range: 0–55 months), respectively. In concordance with current clinical guidelines^33^, combination therapy was in this study mainly reserved for younger patients (median age 58 versus 67 years) with worse prognosis based on high serum LDH levels (LDH ≥ 2ULN; 22.0% versus 5.2% in combination therapy versus monotherapy subgroups, respectively) or presence of brain metastases (35.2% versus 10.5% in combination therapy versus monotherapy subgroups, respectively), with the net effect resulting in similar survival of ICI combination versus monotherapy treated patients (PFS: HR = 1.13, 95% CI 0.752–1.69, Cox *P* = 0.561; OS: HR = 1.39, 95% CI 0.871–2.22, Cox *P* = 0.167; Table 1; Supplementary Fig. S1). The clinical benefit rate was 52.2% (*n* = 93), of which then 28% (*n* = 26) showed a complete response. Pretreatment biopsies for molecular analyses were mainly obtained from subcutaneous tissue (43.3%) and lymph nodes (44.4%). Biopsy location was not associated with response to ICI (Supplementary Fig. S2).

**Table 1:** Baseline characteristics of patients with metastatic melanoma treated with systemic anti-PD-1 monotherapy versus anti-PD-1/ani-CTLA-4 combination therapy.

	Characteristics	Monotherapy (*n* = 124)	Combination therapy (*n* = 54)	*P*-value
Age (years)	Mean (SD)	64 (13)	57 (13)	** < 0.001**
	Missing N (%)	7 (5.6%)	3 (6%)	
Sex N (%)	Female	53 (43%)	22 (41%)	0.93
	Male	71 (57%)	32 (59%)	
	Missing	0 (0%)	0 (0%)	
ECOG PS N (%)	0	82 (66%)	37 (69%)	0.32
	1	34 (27%)	12 (22%)	
	≥ 2	1 (0.81%)	2 (3.7%)	
	Missing	7 (5.6%)	3 (5.6%)	
Confirmed brain metastases N (%)	No	65 (52%)	25 (46%)	**0.0029**
	Yes	13 (10%)	19 (35%)	
	Missing	46 (37%)	10 (19%)	
Liver metastases N (%)	No	73 (59%)	26 (48%)	**0.013**
	Yes	20 (16%)	20 (37%)	
	Missing	31 (25%)	8 (15%)	
Lung metastases N (%)	No	46 (37%)	22 (41%)	1
	Yes	44 (35%)	22 (41%)	
	Missing	34 (27%)	10 (19%)	
Lymph node metastases N (%)	No	18 (15%)	7 (13%)	0.74
	Yes	72 (58%)	37 (69%)	
	Missing	34 (27%)	10 (19%)	
Bone metastases N (%)	No	69 (56%)	33 (61%)	1
	Yes	21 (17%)	11 (20%)	
	Missing	34 (27%)	10 (19%)	
Hemoglobulin (mmol/L)	Mean (SD)	8.8 (0.98)	8.5 (1)	0.12
	Missing N (%)	9 (7%)	3 (6%)	
Neutrophils (10e9/L)	Mean (SD)	5.2 (2)	5.8 (3.1)	0.47
	Missing N (%)	28 (23%)	6 (11%)	
Lymphocytes (10e9/L)	Mean (SD)	4.8 (20)	1.5 (0.55)	0.077
	Missing N (%)	74 (60%)	22 (41%)	
Neutrophil-lymphocyte ratio (NLR)	Mean (SD)	3.5 (1.9)	4.3 (3)	0.29
	Missing N (%)	78 (63%)	22 (41%)	
Tumor marker S100 (µg)	Mean (SD)	0.45 (0.96)	0.55 (1.4)	0.23
	Missing N (%)	67 (54%)	20 (37%)	
Albumin (g/L)	Mean (SD)	42 (4.9)	40 (6.7)	0.09
	Missing N (%)	39 (31%)	11 (20%)	
Lactate dehydrogenase (LDH) (U/I)	Mean (SD)	368 (339)	247 (153)	**0.0032**
	Missing N (%)	9 (7%)	4 (7%)	

Significant values are in bold.

Data are presented as mean ± SD, median [interquartile range] number of patients (%). Abbreviations: ECOG PS, Eastern Cooperative Oncology Group performance status; SD, standard deviation; ULN, upper limit of normal; LNN, lower limit of normal.

### Biomarker validation of ICI response in metastatic melanoma

**Figure 2:**
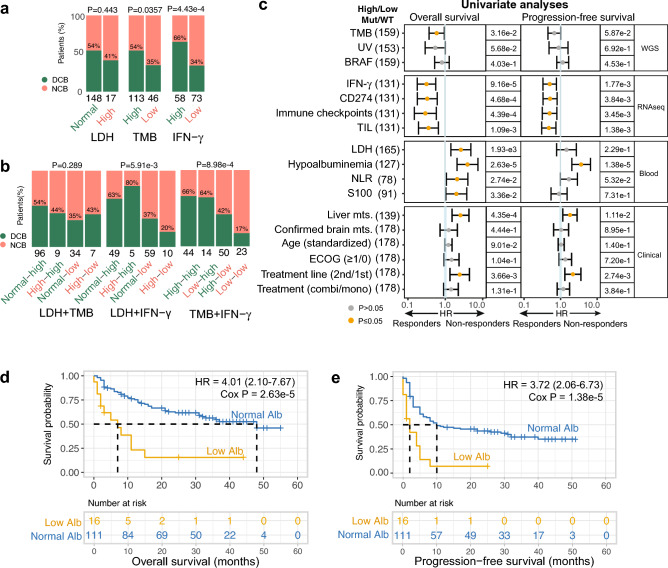
Albumin is a strong predictor for ICI outcome. Shown are (**a**) bar graphs with DCB rate (%) for high versus normal serum LDH or high versus low TMB, and IFN-γ signature expression as standalone biomarkers using Fisher’s exact test or (**b**) combined using Pearson’s chi-squared test. (**c**) Univariate Cox PH regression analyses of predictors for OS and PFS in ICI-treated patients with metastatic melanoma. On the right side, the table depicts the number of patients in the analysis and the associated Cox P for OS and PFS. Each row represents a biomarker and is separated into groups for clinical, WGS, RNA-seq, and blood modalities. Significant P ≤ 0.05 (orange), 95% confidence interval. Estimated OS (**d**) and PFS (**e**) according to serum albumin levels in ICI-treated patients with metastatic melanoma. Survival curves are calculated using the Cox regression PH method. Abbreviations: Alb, albumin; *BRAF*, v-Raf murine sarcoma viral oncogene homolog B1; combi, combination therapy; ECOG PS, Eastern Cooperative Oncology Group performance status; HR, hazard ratio; IC, immune checkpoints; IFN-γ, interferon-gamma; LDH, lactate dehydrogenase; mono, monotherapy; MUT, mutation; mts., metastasis; NLR, neutrophil-lymphocyte-ratio; PH, proportional hazard; RNA-seq, RNA sequencing; S100, serum S100 melanoma marker; TIL, tumor-infiltrating lymphocytes; TMB, tumor mutational burden; UV sig., ultraviolet mutational signature; WGS, whole-genome sequencing.

This study evaluates previously established biomarkers from various modalities, including clinical, genomic, transcriptomic, and blood-based markers (assessed pretreatment), to investigate their association with treatment outcomes in metastatic melanoma. We showed that the three well-established markers, namely LDH (≥ 2ULN), IFN-γ signature expression, and TMB, provided insufficient specificity in identifying (non-)responsive patients as standalone biomarkers (Fig. 2a). Notably, the results demonstrated that among patients with unfavorable biomarker profiles, at least one-third still showed DCB. While pairwise combinations of these biomarkers did show some improvement in patient stratification, these combinations still resulted in frequent misclassifications (Fig. 2b). These findings emphasize the urgent need for novel combinatorial (multimodal) biomarker-based strategies to facilitate clinical decision-making. Next, we conducted univariate analyses to investigate various potential biomarkers derived from literature, and their association with survival (Fig. 2c; Supplementary Table S5). The prognostic markers that were evaluated included TMB, UV mutational signature, *BRAF* mutation status, IFN-γ signature expression, CD274 expression, immune checkpoint expression, TIL signature expression, serum LDH (≥ 2ULN [upper limit of normal]), serum albumin (< LLN [lower limit of normal]), serum NLR (≥ ULN), serum S100 (≥ ULN), liver and brain metastasis, age, ECOG PS, treatment line (first and second) and treatment type (mono- and combination therapy). The results confirmed most of the previously reported associations and identified hypoalbuminemia as the strongest predictor of poor outcome for OS (HR = 4.01, 95% CI 2.10–7.67, Cox *P* = 2.63e−05) and PFS (HR = 3.72, 95% CI 2.06–6.73, Cox *P* = 1.38e−05).

Hypoalbuminemia (*n* = 16) was associated with a significantly worse treatment outcome in terms of OS (HR = 4.01, 95% CI 2.10–7.67, Cox *P* = 2.63e−05; Fig. 2d) and PFS (HR = 3.72, 95% CI 2.06–6.73, Cox *P* = 1.38e−05; Fig. 2e). The univariate analyses were repeated exclusively for monotherapy, as the limited size of the combination therapy subgroup precluded separate analysis, and showed that the association of hypoalbuminemia with outcome remained significant in the monotherapy group in analyses of OS (HR = 3.41, 95% CI 1.31–8.84, Cox *P* = 0.0116) and PFS (HR = 4.58, 95% CI 2.00–10.46, Cox *P* = 3.06e−4; Supplementary Fig. S3). The median PFS for patients with hypoalbuminemia was 2 months, while this was 48 months for those with normal albumin. The median OS was 7 months and 48 months for patients with low versus normal albumin, respectively. Due to the retrospective collection of laboratory measurements in this study, albumin values were missing for a notable fraction of patients; importantly, patient subgroups with versus without albumin measurements were highly comparable in terms of baseline characteristics and survival (Supplementary Table S6, Supplementary Fig. S4).

Exploratory analysis on driver mutations showed no significant associations with treatment outcome (Cox *P* ≤ 0.05, FDR-corrected, Supplementary Table S7, Supplementary Fig. S5). Additionally, other genomic features, including the remaining COSMIC mutational signatures^25^, whole-genome duplication, ploidy status, polyclonal proportion, and sequencing-based tumor purity^22^, did not attain statistical significance (Cox *P* ≤ 0.05, FDR-corrected, Supplementary Table S7). Finally, RNA-seq analyses considering the expression of 52 hallmark signatures confirmed only IFN-γ signature expression activation in responding patients (Cox *P* ≤ 0.05, FDR-corrected, Supplementary Fig. S6, Supplementary Table S7). Furthermore, from blood-based measurements, we reported significant effect on OS and PFS for LDH and hypoalbuminemia, white blood cells, and platelets (Cox *P* ≤ 0.05, FDR-corrected, Supplementary Fig. S7).

### Hypoalbuminemia is an independent predictor of poor survival

**Figure 3:**
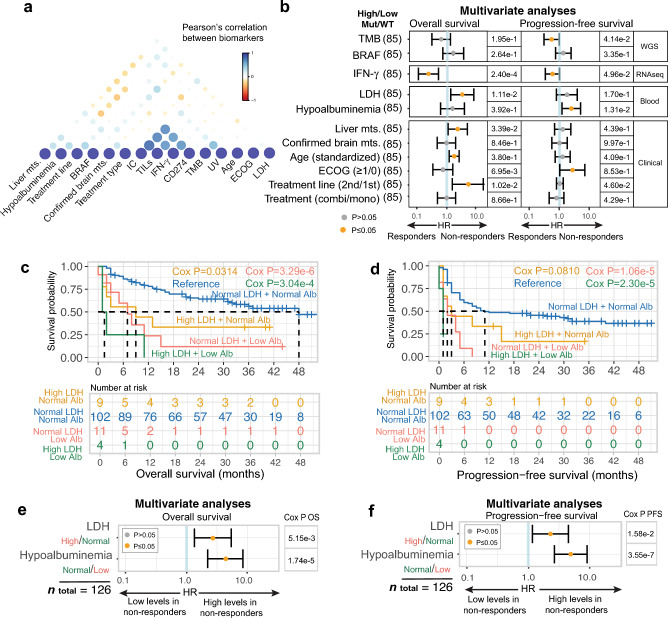
Albumin is an independent biomarker for poor ICI outcome. (**a**) Correlation between biomarkers that are measured on a binary scale. (**b**) Multivariate Cox PH regression analyses of predictors for OS and PFS in ICI-treated patients with metastatic melanoma. On the right side, the table depicts the number of patients in the analysis and the associated Cox P for OS and PFS. Each row represents a biomarker and is separated into groups for clinical, WGS, RNA-seq, and blood modalities. Significant P ≤ 0.05 (orange), 95% confidence interval. Kaplan Meier survival plots stratified by albumin and LDH status for OS (**c**) and PFS (**d**). Survival curves are calculated using the Cox regression PH method. Multivariate Cox PH analysis on LDH and albumin status for OS (**e**) and PFS (**f**). Abbreviations: *BRAF*, v-Raf murine sarcoma viral oncogene homolog B1; combi, combination therapy; ECOG PS, Eastern Cooperative Oncology Group performance status; HR, hazard ratio; IFN-γ, interferon-gamma; LDH, lactate dehydrogenase; mono, monotherapy; MUT, mutation; mts., metastasis; NLR, neutrophil-lymphocyte-ratio; PH, proportional hazard; RNA-seq, RNA sequencing; S100, serum S100 melanoma marker; TMB, tumor mutation burden; UV sig., ultraviolet mutational signature; WGS, whole-genome sequencing.

We then investigated whether serum albumin was an independent predictor from other established biomarkers and clinical factors for the identification of resistance to ICI in patients with metastatic melanoma. In total 85 patients had complete genomic, transcriptomic, blood-based, and clinical data and were included in multivariate analyses. To address biomarker collinearity, only one representative from highly correlated (Pearson *r* > 0.5) biomarker clusters was included (Methods; Fig. 3a). Multivariate Cox PH regression analysis showed that the strongest independent biomarkers for PFS survival were hypoalbuminemia (HR = 2.76, 95% CI 1.24–6.13, Cox *P* = 0.0131), high TMB (HR = 0.506, 95% CI 0.263–0.974, Cox *P* = 0.0414), high IFN-γ signature expression (HR = 0.543, 95% CI 0.296–0.999, Cox *P* = 0.0496) and second treatment line (HR = 3.00, 95% CI 1.02–8.82, Cox *P* = 0.0460), and for OS, high serum LDH (HR = 3.66, 95% CI 1.34–9.96, Cox *P* = 0.0111), high IFN-γ signature expression (HR = 0.195, 95% CI 0.0814–0.466, Cox *P* = 2.40e−04), and treatment line (HR = 6.25, 95% CI 1.55–25.3, Cox *P* = 0.0102; Fig. 3b).

Notably, ECOG PS only explained 5.7% of the variance in hypoalbuminemia and hence could not underlie the prognostic value of hypoalbuminemia in this cohort. Furtheremore, interaction testing using Cox proportional hazards regression showed that the association of hypoalbuminemia with survival was not significantly different in subgroups receiving mono- versus combination therapy (OS: Cox *P* = 0.0175, PFS: Cox *P* = 2.335e−04). The multivariate analyses were repeated exclusively for the subgroup treated with monotherapy (but not for combination therapy only given the limited subgroup size), which comfirmed that hypoalbuminemia was still significantly associated with PFS in the monotherapy group (HR = 3.51, 95% CI 1.28–9.62, Cox *P* = 0.0147; Supplementary Fig. S8). Taken together, these findings show that hypoalbuminemia is associated with poor survival in ICI-treated patients with metastatic melanoma, independently from other biomarkers and clinical factors and could therefore potentially complement a broad set of established biomarkers.

In current clinical practice, the LDH serum level is the only biomarker used guiding or intensifying ICI treatment in metastatic melanoma. Therefore, we specifically investigated the added value of considering hypoalbuminemia in addition to elevated LDH levels (*n* = 126 with both measurments available; Fig. 3c,d). Compared to the subgroup of patients with both an normal LDH and normal albumin (*n* = 102), we observed significantly shorter OS and PFS for patients with hypoalbuminemia and normal LDH (albumin < LLN + LDH < 2ULN, OS: Cox *P* = 3.29e−6, PFS: Cox *P* = 1.06e−5, *n* = 11; Fig. 3c,d), and particularly poor survival in the small subset of patients with hypoalbuminemia plus a high LDH (albumin < LLN + LDH ≥ 2ULN, OS: Cox *P* = 3.04e−4, PFS: Cox *P* = 2.3e−05, *n* = 4; Fig. 3c,d). Furthermore, patients with normal albumin and high LDH levels (≥ 2ULN) showed significantly shorter OS but not PFS as compared to the reference population with normal albumin and normal LDH (OS: Cox *P* = 0.0314, PFS: Cox *P* = 0.0810, *n* = 9; Fig. 3c,d). Importantly, multivariate analysis showed that hypoalbuminemia was also a strong prognostic factor when considered in conjunction with elevated LDH serum levels for both OS (albumin: HR = 4.33, 95% CI 2.22–8.45, Cox *P* = 1.74e−05, LDH: HR = 2.70; 95% CI 1.35–5.43, Cox *P* = 5.15e−03, Fig. 3e) and PFS (albumin: HR = 4.91 95% CI 2.66–9.06, Cox *P* = 3.55e−07; LDH: HR = 2.29, 95% CI 1.17–4.50, Cox *P* = 1.58e−2, Fig. 3f). Taken together, these analyses suggest that hypoalbuminemia is a strong prognostic biomarker for poor outcome of PD-1 blockade in metastatic melanoma, which holds independent prognostic value when considered in conjunction with elevated LDH levels.

### Validation of hypoalbuminemia as a systemic predictor of ICI outcome

**Figure 4:**
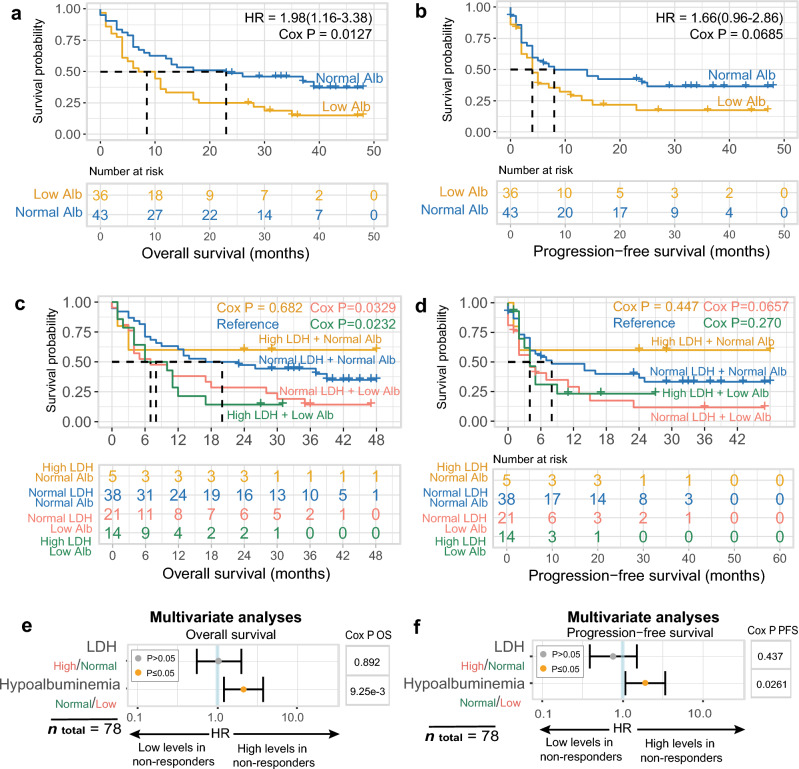
Validation of hypoalbuminemia as an independent predictor to LDH for poor survival. Estimated OS (**a**) and PFS (**b**) according to serum albumin levels in ICI-treated patients with metastatic melanoma. Survival curves are calculated using the Cox regression PH method. Kaplan Meier survival plots stratified by albumin and LDH status for OS (**c**) and PFS (**d**). Multivariate Cox PH analysis on LDH and albumin status for OS (**e**) and PFS (**f**). Abbreviations: Alb, albumin; HR, Hazard ratio; ICI, immune-checkpoint inhibitor; LDH, lactate dehydrogenase; PH, proportional hazard; OS, overall survival; PFS, progression-free survival.

To validate that hypoalbuminemia is an independent predictor for ICI treatment outcome, we collected data of an independent clinical cohort comprising patients with metastatic melanoma (*n* = 79), who received mono- (nivolumab, *n* = 15; pembrolizumab, *n* = 17) or combination therapy (nivolumab plus ipilimumab,* n* = 47), of whom nearly half of them were classified as patients with hypoalbuminemia (*n* = 36; Supplementary Tables S8-S9). Our findings further reinforced our earlier results, indicating that pretreatment hypoalbuminemia is a valuable prognostic indicator for OS (HR = 1.98, 95% CI 1.16–3.38; Cox *P* = 0.0127; Fig. 4a). Patients with hypoalbuminemia had a median OS of 7 months, whereas those with normal albumin had a median OS of 20 months. The analysis of PFS was only near-significant in this cohort (HR = 1.66, 95% CI 0.962–2.86; Cox *P* = 0.0685; Fig. 4b), although patients with hypoalbuminemia had a median of 4 months, while patients with normal albumin had a median PFS of 8 months.

Subsequently, we evaluated the impact of serum albumin status on survival outcomes in relationship with LDH status (*n* = 78; Fig. 4c). Our analyses revealed that compared to the group of patients with normal LDH and normal albumin as reference (*n* = 38), individuals with hypoalbuminemia and high or low LDH exhibited significantly shorter survival OS (albumin < LLN + LDH ≥ 2ULN, Cox *P* = 0.0232, *n* = 14; albumin < LLN + LDH < 2ULN, Cox *P* = 0.0329, *n* = 21). Notably, the subset of patients with normal albumin and high LDH levels (≥ 2ULN) was small (*n* = 5), precluding any definitive conclusions. Although similar trends were observed for PFS, no significant differences were found (Fig. 4d).

Lastly, we investigated the independent effects of hypoalbuminemia and elevated LDH serum levels in a multivariate model and found that only hypoalbuminemia was significantly associated with poor OS (HR = 2.12, 95% CI 1.2–3.72, Cox *P* = 0.00925; Fig. 4e) and poor PFS (HR = 1.91 95% CI 1.08–3.38, Cox *P* = 0.0261; Fig. 4f). We also studied the relationship between ECOG PS and hypoalbuminemia, but found that ECOG PS only explained 0.028% of the variance in serum albumin levels. Additionally, no significant effect of hypoalbuminemia on survival based on mono- versus combination therapy was observed (Cox P ≤ 0.05).

## Discussion

In this multimodal biomarker study, we identified hypoalbuminemia as a strong prognostic factor for poor survival in patients with melanoma receiving ICI treatment. Interestingly, our findings demonstrate that the prognostic capacity of hypoalbuminemia in this context is independent from other established biomarkers (including: elevated LDH, low IFN-γ signature RNA expression, and low TMB) and prognostic clinical characteristics (including: age, ECOG PS, brain or liver metastases, treatment line, mono/combi-treatment). Analysis of the validation cohort confirmed the significant association of hypoalbuminemia with poor OS, even after adjustments of LDH serum levels. Thus, hypoalbuminemia could be a powerful addition to multimodal biomarker strategies for precision immunotherapy in melanoma. In particular, current clinical guidelines consider serum LDH levels for intensifying ICI treatment in melanoma from mono- to combination therapy. Along these lines, we envision that a similar approach might be valuable in which hypoalbuminemia is considered for intensifying ICI treatment of patients with normal LDH levels, highlighting the need of prospective follow-up studies. Furthermore, given the notable poor outcome of ICI treatment in patients with hypoalbuminemia plus elevated LDH levels, prospective follow-up studies are needed to investigate whether these patients would benefit from prioritizing other treatment modalities over ICI treatment, e.g. BRAF/MEK-targeting agents. Given the accessibility and affordability of serum albumin level assessments in routine clinical care, this biomarker holds great potential as a biomarker to improve personalized ICI treatment.

The precise contribution of albumin in modulating immunity and facilitating limited response to ICI remains yet unclear. It is known that serum albumin levels are widely utilized as clinical parameters for evaluating nutritional status^34^ and systemic inflammation^35^. Albumin also plays a pivotal role in stabilizing chemokines and cytokines that attract immune cells to the tumor site, and insufficient levels may impede the efficacy of immunotherapy^36^. Additionally, serum albumin modulates the pharmacokinetics of monoclonal antibodies employed in ICI by reducing the clearance rate and increasing central volume and distribution^37,38^.

Our results fit into a growing body of literature which positions hypoalbuminemia as a powerful predictor of ICI outcome. A recent pan-cancer study with 1714 patients has shown that hypoalbuminemia predicts poor survival upon ICI treatment, but this work lacked a melanoma-specific analysis placing these associations into the context of other melanoma-specific biomarkers^14^. Multiple studies on NSCLC showed that hypoalbuminemia was associated with poor survival after immunotherapy, which held in a multivariate analysis adjusting for treatment line, prior radiotherapy, NLR, and ECOG PS^39,40^. Another study comparing several laboratory and clinical factors in metastatic melanoma reported that albumin was an independent predictor for immunotherapy response after adjustment for LDH, CRP, NLR, brain metastasis, sex, and age^41,42^.

Limitations of our multi-omic real-world dataset with WGS and RNA-seq data include its clinical heterogeneity and the partial unavailability of pretreatment laboratory measurements. For example, pretreatment albumin measurements were lacking in almost a third of the patients. Importantly, no differences were observed in survival or baseline characteristics between patients where albumin was available or missing. Another limitation of the study was the unavailability of another large multi-omic dataset of patients with melanoma treated with ICI for validation. Further verification of the independent prognostic value of albumin in relation to TMB and IFN-γ requires additional multimodal cohorts. Furthermore, as our real-world dataset lacks a placebo arm, placebo-controlled follow-up studies are needed to distinguish the predictive and prognostic value of albumin.

In conclusion, our multi-omic study of metastatic melanoma demonstrates that pretreatment hypoalbuminemia –together with IFN-γ, TMB, and serum LDH– is a strong and independent determinants of survival among ICI-treated patients with this disease. Therefore, hypoalbuminemia has clear potential as a cost-effective and readily available biomarker for personalized immunotherapy in metastatic melanoma.

### Supplementary Information

Supplementary Information 1.

Supplementary Information 2.

Supplementary Information 3.

Supplementary Information 4.

Supplementary Information 5.

Supplementary Information 6.

Supplementary Information 7.

Supplementary Information 8.

Supplementary Information 9.

Supplementary Information 10.

## Data Availability

The clinical data collected in this study are available within the article and its supplementary data files. Expression and genomic profile data analyzed in this study are available at the Hartwig Medical Foundation database under request (j.vd.haar@nki.nl).
