# Supplementary material for: Multi-omic analysis identifies hypoalbuminemia as independent biomarker of poor outcome upon PD-1 blockade in metastatic melanoma
Source: Sci Rep. 2024 May 16;14:11244. doi: 10.1038/s41598-024-61150-y (PMC11099084; doi:10.1038/s41598-024-61150-y)
Supplement: Supplementary file 10 — Supplementary Information 10. [file 41598_2024_61150_MOESM10_ESM.pdf]

Supplementary figures

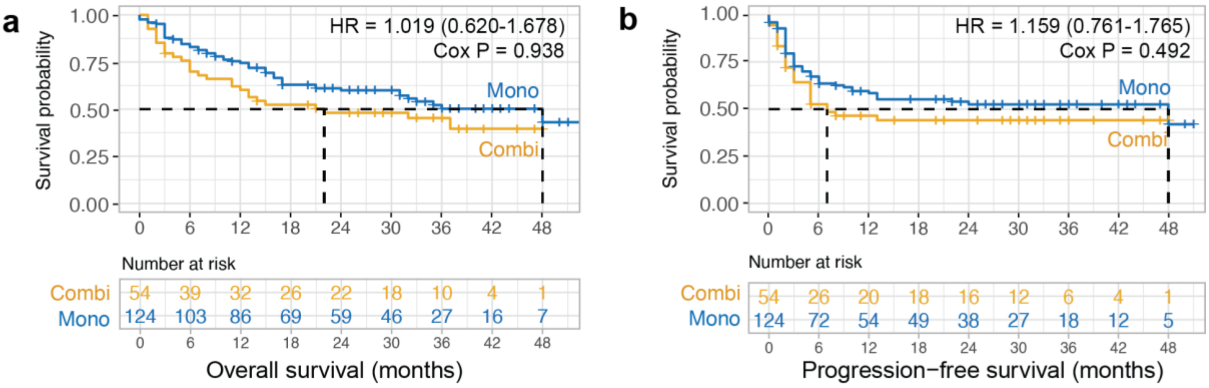

Supplementary Figure 1. Survival of Combination Therapy (CTLA-4/PD-1) versus Monotherapy (PD-1). Kaplan-Meier survival curves depicting the (**a**) overall survival (OS) and (**b**) progression-free survival (PFS) outcomes in patients receiving combination therapy (yellow line) compared to monotherapy (blue line). The x-axis represents time in months, while the y-axis represents the probability of survival.

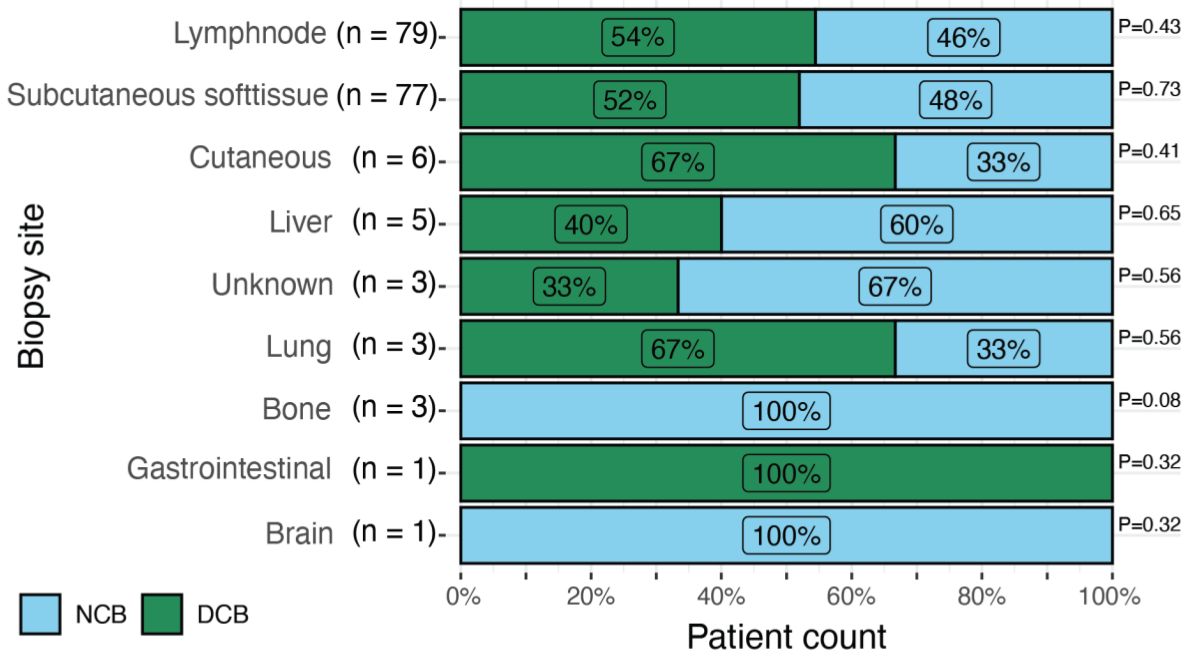

Supplementary Figure 2. Patient Count per Biopsy Tissue Site, Stratified by Clinical Benefit. Stacked barplots illustrating the distribution of patient count across various biopsy tissue sites, categorized by clinical benefit. Each barplot shows the number of patients with durable clinical benefit (DCB) and non-durable clinical benefit (NCB).

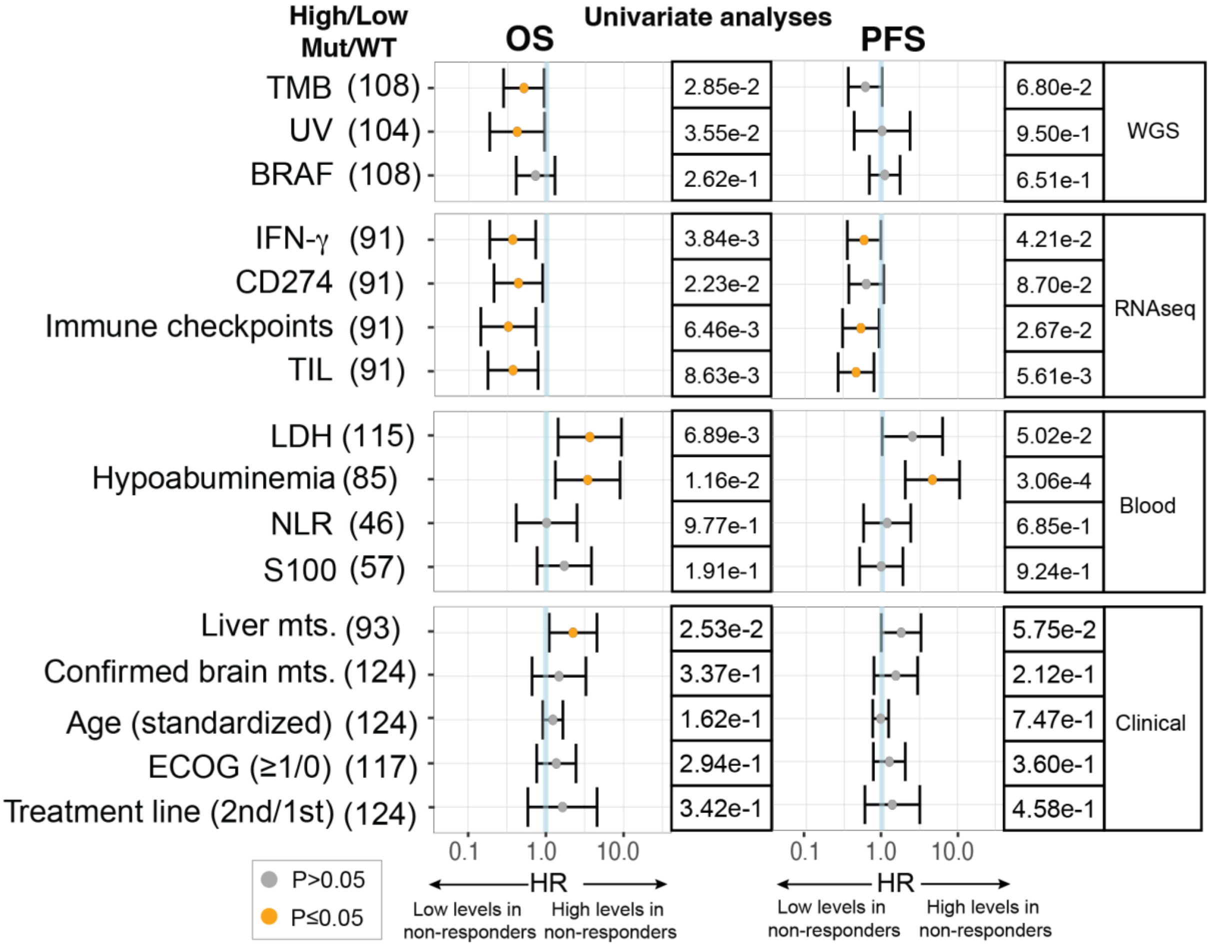

Supplementary Figure 3. Univariate Cox PH analysis on patients receiving monotherapy. Univariate Cox PH regression analyses of predictors for OS and PFS in patients with metastatic melanoma who received monotherapy. On the right side, the table depicts the number of patients in the analysis and the associated Cox P for OS and PFS. Each row represents a biomarker and is separated into groups for clinical, WGS, RNA-seq, and blood modalities. Significant P≤0.05 (orange), 95% confidence interval.

Abbreviations: *BRAF*, v-Raf murine sarcoma viral oncogene homolog B1; combi, combination therapy; ECOG PS, Eastern Cooperative Oncology Group performance status; HR, hazard ratio; IC, immune checkpoints; IFN-γ, interferon-gamma; LDH, lactate dehydrogenase; mono, monotherapy; MUT, mutation; mts., metastasis; NLR, neutrophil-lymphocyte-ratio; RNA-seq, RNA sequencing; S100, serum S100 melanoma marker; TIL, tumor-infiltrating lymphocytes; TMB, tumor mutational burden; UV sig., ultraviolet mutational signature; WGS, whole-genome sequencing.

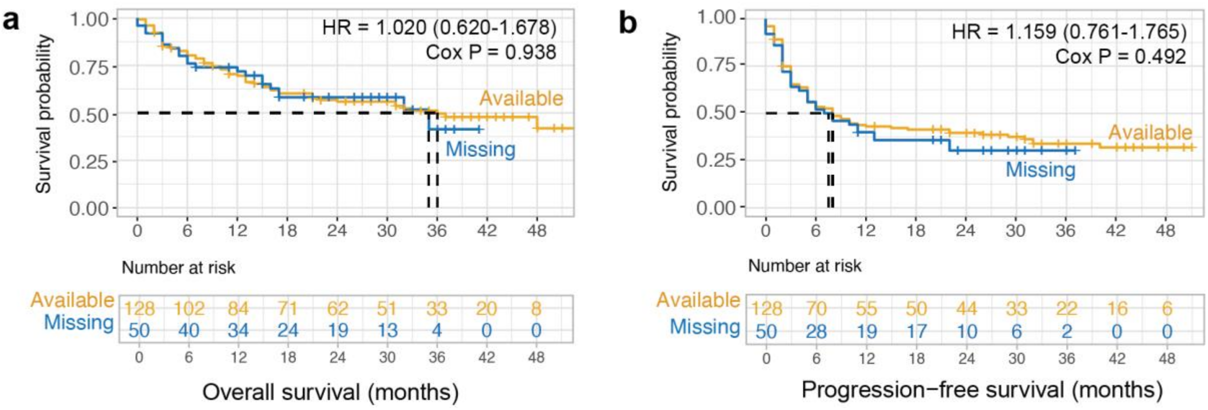

Supplementary Figure 4. Survival of patients with albumin measurements available versus albumin measurement missing. Kaplan-Meier survival curves depicting the (**a**) overall survival (OS) and (**b**) progression-free survival (PFS) outcomes in patients with albumin measurement available (yellow line) compared to missing (blue line). The x-axis represents time in months, while the y-axis represents the probability of survival.

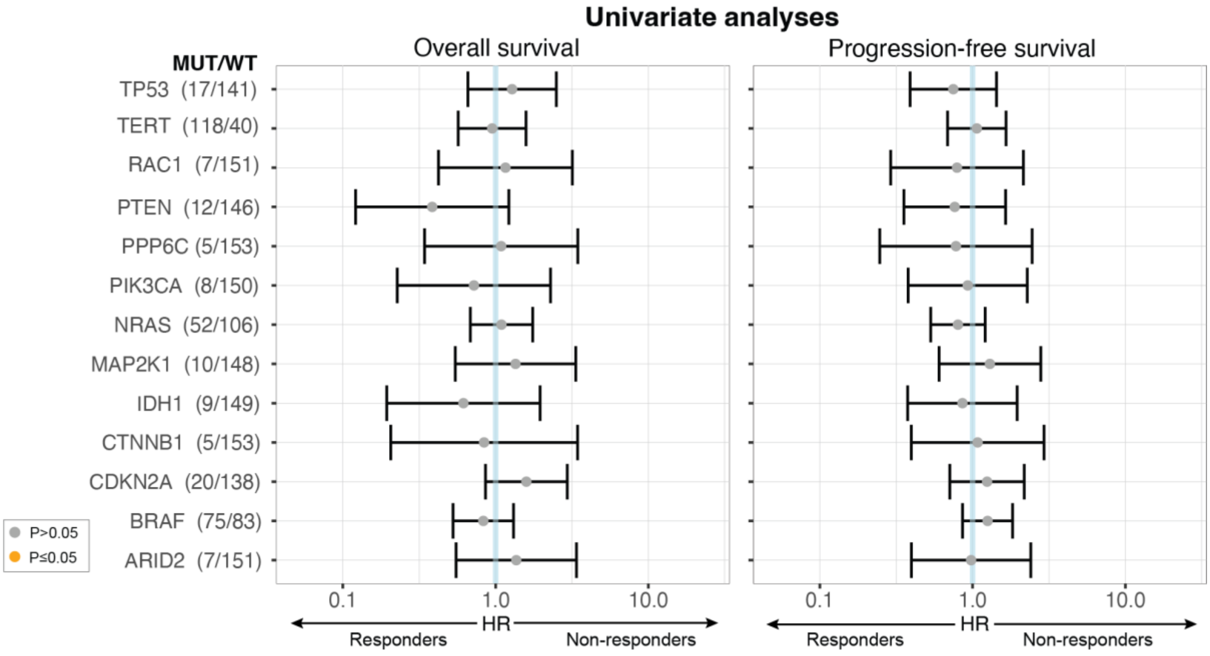

Supplementary Figure 5. Univariate Cox PH regression on prevalent mutations. Forest plots displaying hazard ratios (HR) and 95% confidence intervals (CI) for mutations with a prevalence ≥5 patients (univariate Cox PH regression), with respect to OS and PFS outcomes. No statistically significant associations below a false discovery rate (FDR)-corrected significance threshold of 0.05 are observed.

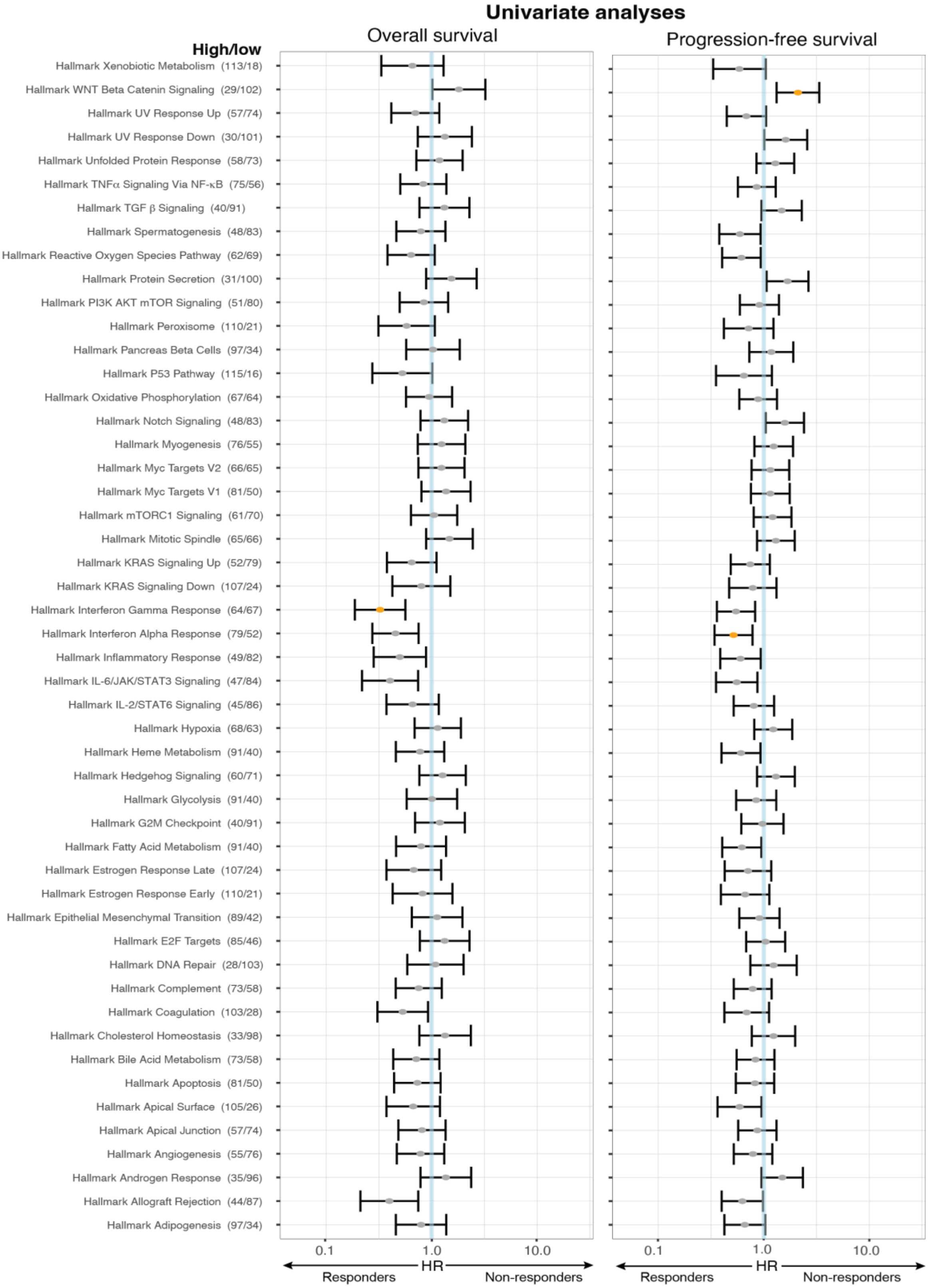

Supplementary Figure 6. Univariate Cox PH regression on gene expression signatures. Forest plots displaying hazard ratios (HR) and 95% confidence intervals (CI) for the hallmark gene signatures (univariate Cox PH regression), with respect to OS and PFS outcomes. Statistically significant associations below a false discovery rate (FDR)-corrected significance threshold of 0.05 are indicated in orange.

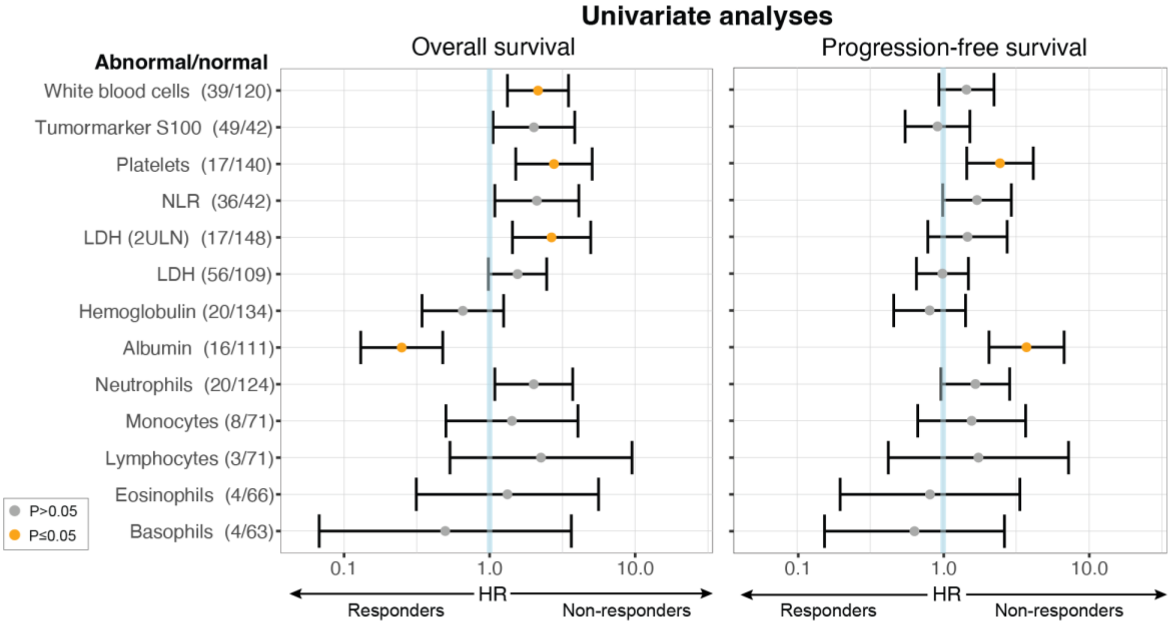

Supplementary Figure 7. Univariate Cox PH regression on blood-based markers. Forest plots displaying hazard ratios (HR) and 95% confidence intervals (CI) for several blood-based markers (univariate Cox PH regression), with respect to OS and PFS outcomes. Statistically significant associations below a false discovery rate (FDR)-corrected significance threshold of 0.05 are indicated in orange.

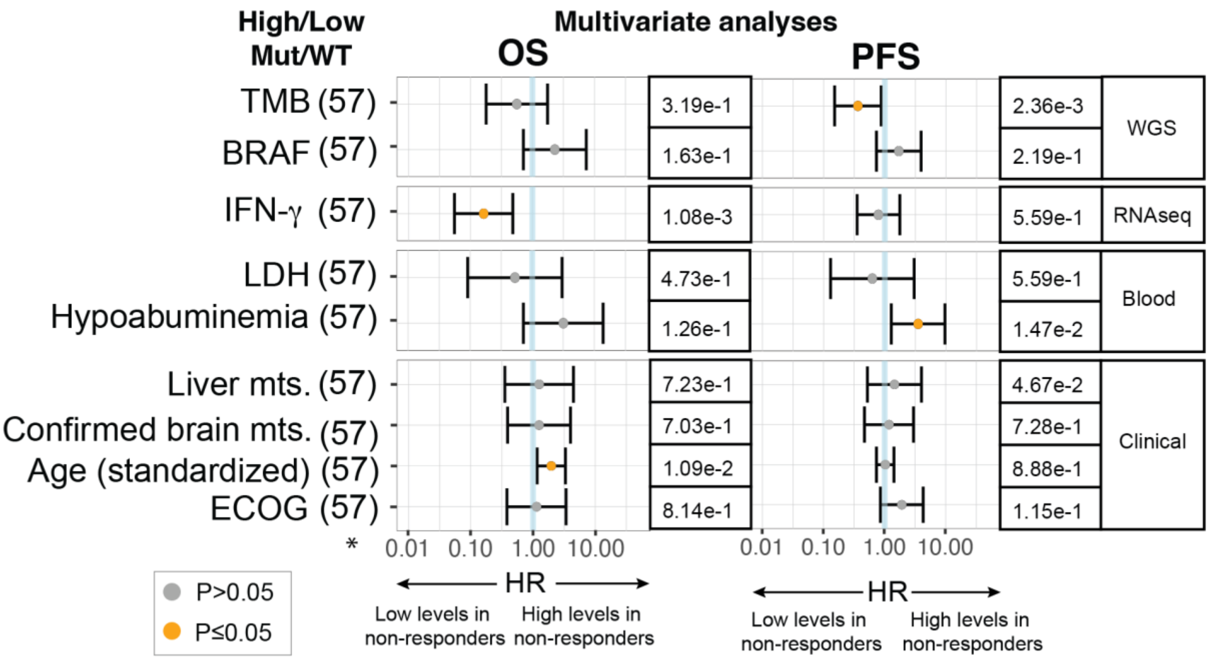

Supplementary Figure 8. Multivariate Cox PH analysis on patients receiving monotherapy. Multivariate Cox PH regression analyses of predictors for OS and PFS in patients with metastatic melanoma who received monotherapy. On the right side, the table depicts the number of patients in the analysis and the associated Cox P for OS and PFS. Each row represents a biomarker and is separated into groups for clinical, WGS, RNA-seq, and blood modalities. Significant P≤0.05 (orange), 95% confidence interval.

*Treatment line was excluded as variable because only one patient received second line therapy.

Abbreviations: *BRAF*, v-Raf murine sarcoma viral oncogene homolog B1; combi, combination therapy; ECOG PS, Eastern Cooperative Oncology Group performance status; HR, hazard ratio; IFN-γ, interferon-gamma; LDH, lactate dehydrogenase; mono, monotherapy; MUT, mutation; mts., metastasis; NLR, neutrophil-lymphocyte-ratio; RNA-seq, RNA sequencing; S100, serum S100 melanoma marker; TIL, tumor-infiltrating lymphocytes; TMB, tumor mutational burden; UV sig., ultraviolet mutational signature; WGS, whole-genome sequencing.
